# The Transcriptome Characterization of the Hypothalamus and the Identification of Key Genes during Sexual Maturation in Goats

**DOI:** 10.3390/ijms251810055

**Published:** 2024-09-19

**Authors:** Qing Li, Tianle Chao, Yanyan Wang, Rong Xuan, Yanfei Guo, Peipei He, Lu Zhang, Jianmin Wang

**Affiliations:** 1 Shandong Provincial Key Laboratory of Animal Biotechnology and Disease Control and Prevention, College of Animal Science and Veterinary Medicine, Shandong Agricultural University, Tai’an 271000, China; l18853852560@163.com (Q.L.); 178266218200@163.com (Y.W.); xuanrong@sdau.edu.cn (R.X.); 2021110328@sdau.edu.cn (Y.G.); 2022110335@sdau.edu.cn (P.H.); ka205486665@163.com (L.Z.); 2 Key Laboratory of Efficient Utilization of Non-Grain Feed Resources (Co-Construction by Ministry and Province), Ministry of Agriculture and Rural Affairs, Shandong Agricultural University, Tai’an 271000, China

**Keywords:** goats, transcriptomics, hypothalamus, serum hormone, reproduction

## Abstract

Sexual maturation in goats is a dynamic process regulated precisely by the hypothalamic–pituitary–gonadal axis and is essential for reproduction. The hypothalamus plays a crucial role in this process and is the control center of the reproductive activity. It is significant to study the molecular mechanisms in the hypothalamus regulating sexual maturation in goats. We analyzed the serum hormone profiles and hypothalamic mRNA expression profiles of female goats during sexual development (1 day old (neonatal, D1, n = 5), 2 months old (prepuberty, M2, n = 5), 4 months old (sexual maturity, M4, n = 5), and 6 months old (breeding period, M6, n = 5)). The results indicated that from D1 to M6, serum hormone levels, including FSH, LH, progesterone, estradiol, IGF1, and leptin, exhibited an initial increase followed by a decline, peaking at M4. Furthermore, we identified a total of 508 differentially expressed genes in the hypothalamus, with a total of four distinct expression patterns. Nuclear receptor subfamily 1, group D, member 1 (*NR1D1*), glucagon-like peptide 1 receptor (*GLP1R*), and gonadotropin-releasing hormone 1 (*GnRH-1*) may contribute to hormone secretion, energy metabolism, and signal transduction during goat sexual maturation via circadian rhythm regulation, ECM receptor interactions, neuroactive ligand–receptor interactions, and Wnt signaling pathways. This investigation offers novel insights into the molecular mechanisms governing the hypothalamic regulation of goat sexual maturation.

## 1. Introduction

Goats are one of the most important domestic animals in the world that provide people with products such as meat, milk, skin, and cashmere [1,2,3]. Reproductive traits are economically important traits in livestock, and the onset of puberty is a critical transition period for animals to achieve sexual maturity and acquire reproductive capacity [4,5]. Elucidating the physiological and molecular mechanisms behind development and sexual maturation in goats is an important guide for breeding practices.

The hypothalamic–pituitary–gonadal (HPG) axis regulates reproductive activity in female mammals. Sexual maturation and other reproductive processes are induced by a complex cascade of neuronal and glial interaction events within the hypothalamus, which are controlled by metabolic as well as genetic and environmental influences (feedback from sex steroids, nutritional status, seasonal/photoperiodic cycles, etc.) [6]. The hypothalamus, located upstream of the HPG axis, is an important neuroendocrine center [7]. It can initiate puberty by modulating endocrine, circadian rhythms, feeding, and gonadal development [8]. Gonadotropin-releasing hormones (GnRHs) released from the hypothalamus act on the pituitary gland to regulate the release of pituitary follicle-stimulating hormones (FSHs) and luteinizing hormones (LHs), which act on the ovaries and regulate the production of gonadal steroid hormones, and GnRH release is also regulated by negative feedback from sex hormones [9]. Thus, the hypothalamus plays an important role in mammalian sexual development [10].

In recent years, researchers have extensively investigated the regulation of reproductive capacity within the hypothalamus of domestic animals [11,12,13]. Transcriptomic techniques effectively delineate gene expression patterns within the hypothalamus and other tissues, elucidating the underlying mechanisms governing reproductive and sexual development. A study examining hypothalamic tissue from goats with high and low fertility levels yields novel insights into the molecular pathways through which the hypothalamus modulates fecundity [14]. Alterations in hypothalamic gene expression pre- and post-puberty in sheep impact the onset of puberty [15]. Furthermore, the expression of E2F Transcription Factor 8 (*E2F8*), Nuclear Factor of Activated T Cells 5 (*NFAT5*), and SIX Homeobox 5 (*SIX5*) in the hypothalamus of Brahman heifers may exert regulatory effects on puberty [16]. Investigations in sows have also identified key genes potentially implicated in puberty regulation, including Estrogen Receptor 1 (*ESR1*), Neurofibromin 1 (NF1), and Amyloid Beta Precursor Protein (*APP*) [17]. These findings underscore the intricate changes in hypothalamic transcriptomic profiles during sexual development in livestock.

The Jining grey goat is a prominent local goat breed in China, renowned for its early sexual maturity, strong fecundity, and year-round estrus [18,19]. The female Jining grey goat can attain puberty as early as 2 months of age and can reach sexual maturity at 3–4 months, with an estrous cycle of approximately 18 days [20,21]. These characteristics make it an ideal model for investigating the reproductive physiology of domestic animals. However, most studies on the hypothalamus of goats have focused on pre- and post-pubertal comparisons, which fail to fully characterize the complete sexual maturation process of goats after birth [22,23,24]. Additionally, these studies often employed a limited number of biological replicates. Therefore, to address these gaps, we investigated the hypothalamic tissue expression profile of the female Jining grey goat at 1 day old (neonatal, D1, n = 5), 2 months old (pre-puberty, M2, n = 5), 4 months old (sexual maturity, M4, n = 5), and 6 months old (breeding period, M6, n = 5) to identify key genes and pathways regulating sexual development from birth to sexual maturity in goats. These findings offer a more comprehensive understanding of the dynamic changes in mRNA during sexual maturation, thereby revealing the molecular regulatory mechanisms underlying goat sexual maturation.

## 2. Results

### 2.1. Analysis of Serum Hormone Index

**Figure 1:**
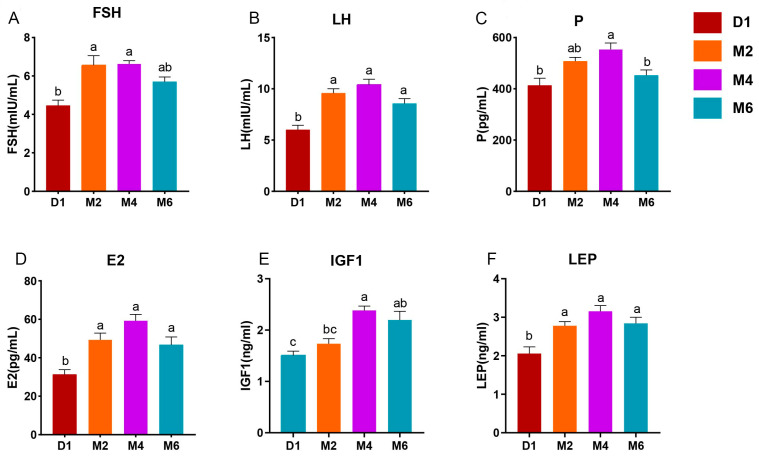
Changes in serum hormone levels in Jining grey goats during sexual maturation. (**A**). Follicle-stimulating hormone, FSH. (**B**). Luteinizing hormones, LH. (**C**). Progesterone, P. (**D**). Estradiol, E2. (**E**). Insulin-like growth factor 1, IGF-1. (**F**). Leptin, LEP. Different lowercase letters indicate significant differences in phenotypic indicators between different days of age (*p* < 0.05). All data are presented as mean ± standard error. D1, M2, M4, and M6 represent 1-day-old, 2-month-old, 4-month-old, and 6-month-old goats, respectively.

This study investigated the dynamic changes in endocrine hormones during goat sexual development by measuring the levels of FSH, LH, progesterone (P), estradiol (E2), insulin-like growth factor 1 (IGF-1), and Leptin (LEP) in goat serum from the D1 to M6 period (Figure 1A–F). The results revealed varied expression patterns of hormone levels in the serum of Jining grey goats. Specifically, the serum levels of FSH, LH, E2, and LEP were significantly higher at M2 and M4 compared to D1 (*p* < 0.05). Additionally, there were no significant differences in the levels of FSH, LH, E2, and LEP among the M2, M4, and M6 periods (*p* > 0.05). Notably, all hormone levels were lowest during the D1 and peaked during the M4. Furthermore, correlation analysis between body weight and hormone levels revealed significant positive correlations between FSH, LH, and P (*p* < 0.05) (Figure S1). Similarly, E2, IGF-1, and LEP exhibited significant positive correlations with LH (*p* < 0.05).

### 2.2. Overview of RNA-Seq

**Figure 2:**
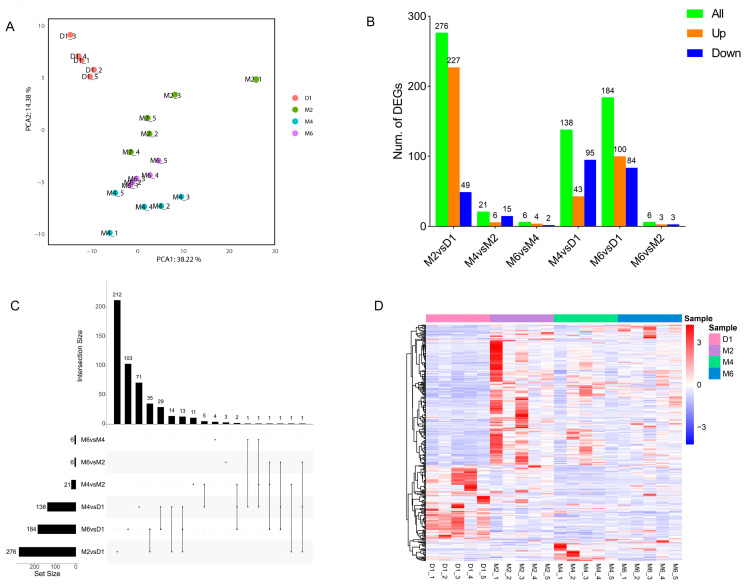
Overview of transcriptome analysis based on FPKM values at four stages of goat hypothalamus development. (**A**). PCA of 20 hypothalamic transcriptome data at four developmental stages of goats. (**B**). Histogram of the number of DEGs. (**C**). UpSet plots of the DEG. (**D**). The clustering heatmap of the DEGs in the 20 samples.

To study the transcriptome dynamics during sexual maturation in goats, RNA-seq sequencing was performed on hypothalamic tissues from goats of different ages (D1, M2, M4, M6); a total of 264.61 G clean reads were obtained, with an effective data volume of 12.35–14.14 G per sample, and the percentage of Q30 ranged from 90.4% to 93.56%, with an average GC content of 44.98% (Table S1). The sequence alignment of clean reads to the goat reference genome using HISAT2 showed alignment rates of 86.05–95.99% (Table S2), indicating the better quality and splicing results of the sequencing data. The fragments per kilobase of exon model per million mapped fragments (FPKM) obtained after matching with the reference genome were subjected to principal component analysis (PCA) and cluster analysis to observe the correlation between samples and the reliability of the experimental data. The PCA results indicate that D1 is distinctly separated from the other three sample groups, while the distance between M4 and M6 is closer (Figure 2A), suggesting that as goat sexual development matures, the physiological differences in goat hypothalamic tissue gradually decrease.

### 2.3. Identification of the DEGs

By comparing the hypothalamic tissue of the four developmental stages in pairs, in the six comparison groups (M2 vs. D1, M4 vs. D1, M6 vs. D1, M4 vs. M2, M6 vs. M2, M6 vs. M4), a total of 508 differentially expressed genes (DEGs) were identified (Figure 2B, Tables S3–S9). Among these, the largest number of DEGs was observed between M2 and D1, with a total count of 276 (227 upregulated, 49 downregulated). In the M4 vs. M2 comparison group, there were twenty-one DEGs (six upregulated, five downregulated). The M6 vs. M4 group exhibited a total of six DEGs (four upregulated, four downregulated). For the M4 vs. D1 group, we identified one hundred and thirty-eight DEGs (three upregulated, ninety-five downregulated). There were 184 DEGs in the M6 vs. D1 group (100 upregulated, 95 downregulated). Finally, only six DEGs (three upregulated, three downregulated) were found in the M6 vs. D1.

UpSet plots showed (Figure 2C) that there were two hundred and twelve independently expressed differential genes in M2 vs. D1, four independently expressed differential genes in M6 vs. M4, and eleven independently expressed differential genes in M4 vs. M2. Furthermore, a total of 13 DEGs were identified across the comparison groups M2 vs. D1, M4 vs. D1, and M6 vs. D1. The results of the clustering analysis of DEGs indicated that the three groups of M2, M4, and M6 had similar expression patterns, while the goats in D1 were more different from the other three age groups (Figure 2D). There were obvious differences in the expression patterns of differential genes at different developmental stages.

### 2.4. Functional Enrichment Analysis of DEGs

**Figure 3:**
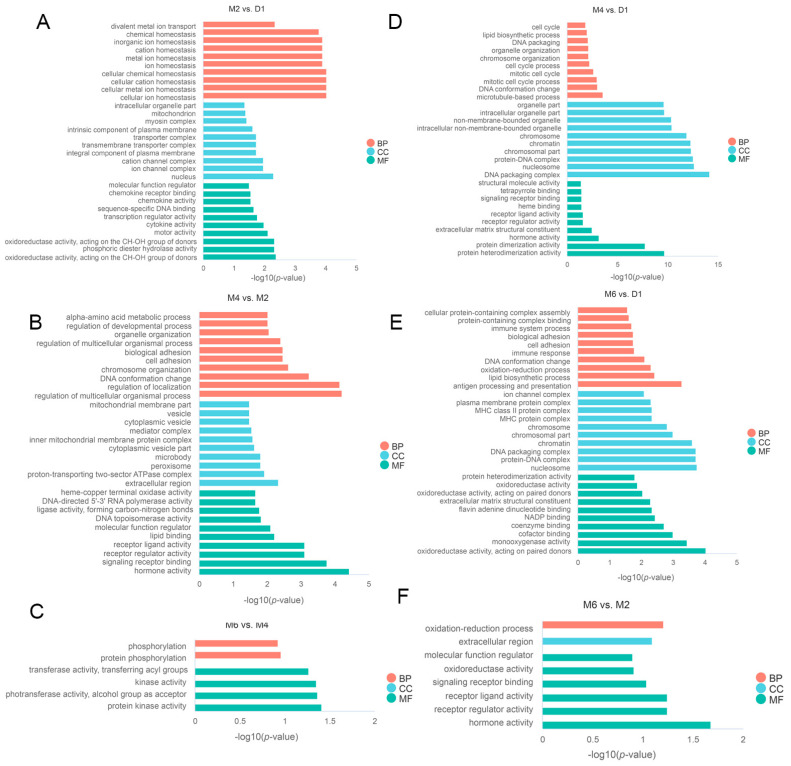
Go enrichment analysis of DEGs in different comparison groups during sexual maturation in Jining grey goats. The results of GO enrichment analysis for M2 vs. D1 (**A**), M4 vs. D1 (**B**), M4 vs. M2 (**C**), M6 vs. D1 (**D**), M6 vs. M4 (**E**), and M6 vs. M2 (**F**) are presented respectively. BP, biological process; CC, cellular component; MF, molecular function.

**Figure 4:**
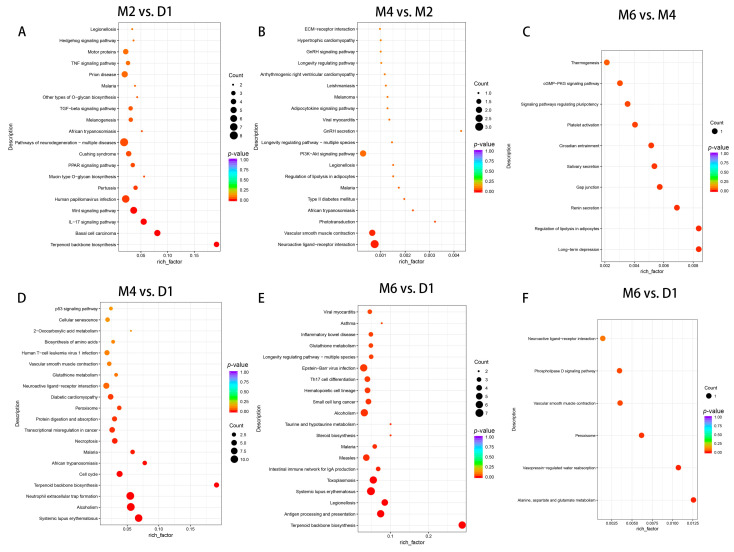
KEGG enrichment analysis of DEGs in different comparison groups during sexual maturation in Jining grey goats. (**A**) M2 vs. D1, (**B**) M4 vs. M2, (**C**) M6 vs. M4, (**D**) M4 vs. D1, and (**E**,**F**) M6 vs. D1.

To gain deeper insights into the functional roles of DEGs during the sexual maturation process of goats, we conducted Gene Ontology (GO) and Kyoto Encyclopedia of Genes and Genomes (KEGG) enrichment analyses on DEGs identified across various comparative groups (M2 vs. D1, M4 vs. D1, M6 vs. D1, M4 vs. M2, M6 vs. M2, M6 vs. M4) (Figure 3A–F and Figure 4A–F).

The results revealed the significant enrichment of GO terms in the comparison of M2 vs. D1, including cellular ion homeostasis, mitotic cell cycle, transmembrane transport, and G protein-coupled receptor binding (*p* < 0.05). In M4 vs. D1, GO terms such as the cell cycle process, the cellular lipid metabolic process, and hormone activity were significantly enriched (*p* < 0.05). In M6 vs. D1, enriched GO terms included antigen processing and presentation, lipid biosynthetic processes, oxidation-reduction processes, and immune system processes (*p* < 0.05). In the M4 vs. M2 comparison, significantly enriched GO terms comprised hormone activity, signaling receptor binding, and lipid binding (*p* < 0.05). However, in M6 vs. M2, no significant enrichment was observed in GO terms; in M6 vs. M4, protein kinase activity and kinase activity GO terms were significantly enriched (*p* < 0.05).

Based on the KEGG enrichment analysis, in the comparison of M2 vs. D1, a total of 15 pathways were identified, which included terpenoid backbone biosynthesis, basal cell carcinoma, the IL-17 signaling pathway, the Wnt signaling pathway, the PPAR signaling pathway, and the TGF-beta signaling pathway (*p* < 0.05). In the comparison of M4 vs. D1, pathways such as neutrophil extracellular trap formation, cell cycle, and protein digestion and absorption were significantly enriched (*p* < 0.05). Similarly, in the comparison of M6 vs. D1, a total of 29 significantly enriched KEGG pathways were identified, which included antigen processing and presentation, the intestinal immune network for IgA production, steroid biosynthesis, glutathione metabolism, and the serotonergic synapse (*p* < 0.05). In comparisons between M4 and M2, as well as M6 and M2, the KEGG pathways were less enriched. Only four pathways were significantly enriched in M4 vs. M2, including neuroactive ligand–receptor interaction and vascular smooth muscle contraction (*p* < 0.05). In M6 vs. M2, the Alanine, aspartate and glutamate metabolism, Vasopressin-regulated water reabsorption, vascular smooth muscle contraction, and Phospholipase D signaling pathways were significantly enriched (*p* < 0.05). Although KEGG pathways are enriched in all of these comparison groups, the number of significantly enriched pathways and the number of genes enriched in the pathways are relatively low, which may mask critical information concerning the dynamic changes in gene expression during the developmental process of goats.

### 2.5. Expression Patterns and Functional Analysis of DEGs Identified during Sexual Maturation

**Figure 5:**
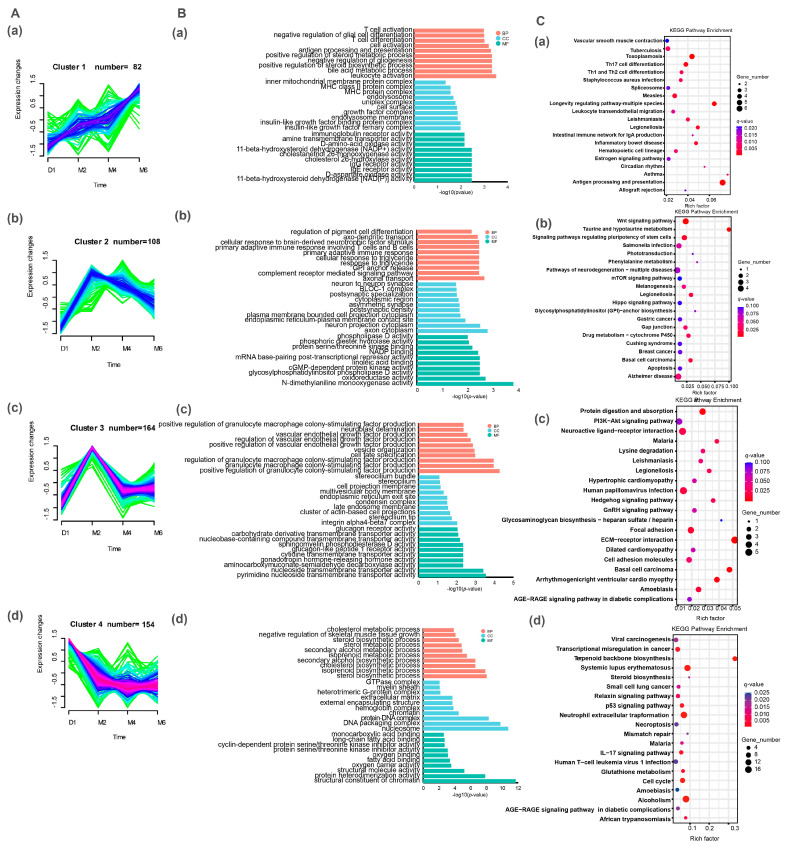
Expression pattern cluster analysis based on DEGs. (**A**): (**a**–**d**) Four different expression patterns of DEGs identified using Mfuzz. (**B**): (**a**–**d**) Results of GO analysis in the 4 clusters. (**C**): (**a**–**d**) Results of KEGG enrichment of the top 20 DEGs in the 4 clusters.

In order to further investigate the functional roles of the 508 DEGs identified in the hypothalamus during the sexual maturation process of goats, we first conducted an analysis of their expression patterns. The results show (Figure 5A, Table S10) that these 508 genes can be classified into four different expression patterns. Cluster 1 contained 82 DEGs, which had the lowest expression level in the D1 stage and gradually increased with age, reaching a peak in the M6 stage. The expression level of 108 DEGs in cluster 2 was still the lowest in the D1 stage and showed an obvious increasing trend in the M2 stage, then decreased gradually from M2 to M6. Cluster 3 contained the highest number of DEGs, totaling 164, with a marked increase in expression levels from D1 to M2, followed by a clear decrease from M2 to M4. The expression level of 154 DEGs in cluster 4 was the highest at the D1 stage, followed by a distinct decrease at M2, and a slight additional decrease from M2 to M6 stages.

DEGs with different expression patterns may play a similar role during sexual maturation in goats. Furthermore, we performed GO and KEGG enrichment analyses on these four clusters of DEGs with different expression patterns, respectively (Figure 5B,C, Tables S11–S18). GO analysis showed that DEGs in cluster 1 were significantly enriched in the positive regulation of leukocyte activation, bile acid biosynthesis, and steroid biosynthesis (*p* < 0.05). KEGG results showed that these DEGs were significantly enriched in immune, reproductive, and energy metabolism-related pathways, such as Th17 cell differentiation, Th1 and Th2 cell differentiation, circadian rhythm, and the estrogen signaling pathway (*p* < 0.05). DEGs in cluster 2 were significantly enriched in GO items related to neural signal transduction, such as axonal transport, complement receptor-mediated signaling pathways, and neuronal neurite cytoplasm (*p* < 0.05). The results of KEGG enrichment analysis showed that the Wnt signaling pathway, the signaling pathway regulating stem cell pluripotency, and the drug metabolism-cytochrome P450 pathway were significantly enriched in cluster 2 (*p* < 0.05). GO analysis showed that DEGs in cluster 3 were significantly enriched in GO entries, such as the positive regulation of vascular endothelial growth factor production and neuroblast stratification (*p* < 0.05). KEGG analysis showed that the ECM–receptor interaction, protein digestion and absorption, neuroactive ligand–receptor interaction, hedgehog signaling, cell adhesion molecule, and lysine degradation pathways were significantly enriched in cluster 3 (*p* < 0.05). DEGs in cluster 4 were significantly enriched in steroid biosynthesis, lipid biosynthesis, skeletal muscle tissue growth, and calcium ion binding (*p* < 0.05). These DEGs were significantly enriched in pathways related to immunity, substance synthesis and metabolism, signal transduction, and cell fate, such as the IL-17 signaling pathway, glutathione metabolism, the relaxin signaling pathway, steroid biosynthesis, apoptosis, cell cycle, and the p53 signaling pathway in cluster 4 (*p* < 0.05).

To identify key genes involved in sexual maturation in goats, we conducted a correlation analysis between DEGs significantly enriched in the reproduction-associated KEGG pathway and serum hormone levels (Figure S2). Among the 47 DEGs analyzed, glucagon-like peptide 1 receptor (*GLP1R*), catenin delta 2 (*CTNND2*), and gonadotropin-releasing hormone 1 (*GnRH1*) showed significant positive correlations with FSH levels (*p* < 0.05). In addition, *CTNND2* and peroxisome proliferator-activated receptor delta (*PPARD*) were significantly positively correlated with various hormones such as FSH, LH, P, and LEP (*p* < 0.05).

### 2.6. PPI Network Analysis

**Figure 6:**
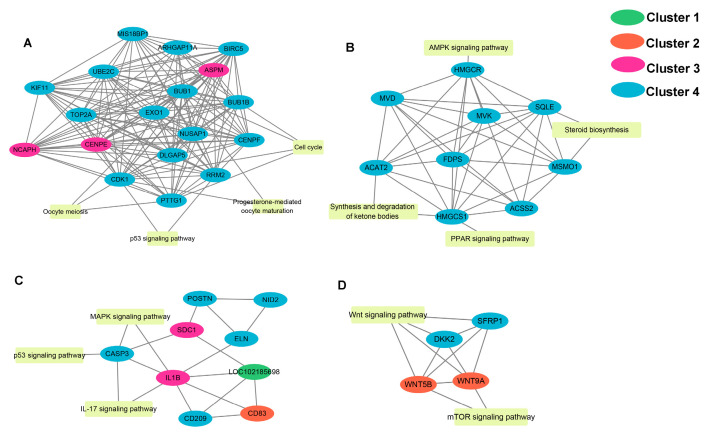
PPI network modules within the hypothalamus. Four significant modules were constructed using the PPI network of DEGs. (**A**): Module 1 (MCODE score = 17.89) (**B**): Module 2 (MCODE score = 9.56) (**C**): Module 2 (MCODE score = 4) (**D**): Module 4 (MCODE score = 4). Different colors represent DEGs with different modes of expression.

To identify key genes involved in sexual maturation in goats, we mapped the protein–protein interaction (PPI) network of 508 DEGs identified in the hypothalamus of goats at different developmental stages. The network consists of 267 nodes and 620 edges. Different colors indicate DEGs with different expression patterns. We used five different algorithms in the cytoHubba plugin to filter out the top ten DEGs from the PPI network. We intersected the key genes obtained from these five results and obtained a total of six key genes (Figure S3, Table S19), including BUB1 Mitotic Checkpoint Serine/Threonine Kinase (*BUB1*), Centromere Protein E (*CENPE*), Baculoviral IAP Repeat Containing 5 (*BIRC5*), Kinesin Family Member 11 (KIF11), and DLG-Associated Protein 5 (*DLGAP5*). In addition, by MCODE, we constructed four modules in the PPI network, and KEGG enrichment analysis was performed on the genes within the module (Figure 6A–D). Module 1 comprised 19 significantly enriched DEGs associated with the cell cycle, oocyte meiosis, and the p53 signaling pathway (*p*< 0.05). Module 2 consisted of 10 genes significantly enriched in terpenoid backbone biosynthesis, metabolic pathways, ketone body synthesis and degradation, and steroid biosynthesis (*p* < 0.05). In Module 3, the 10 DEGs were significantly enriched in the IL-17, MAPK, and TNF signaling pathways (*p* < 0.05). Module 4 contained only four genes, which were significantly enriched in the Wnt signaling pathway (*p* < 0.05).

### 2.7. qRT-PCR Validation of DEGs

**Figure 7:**
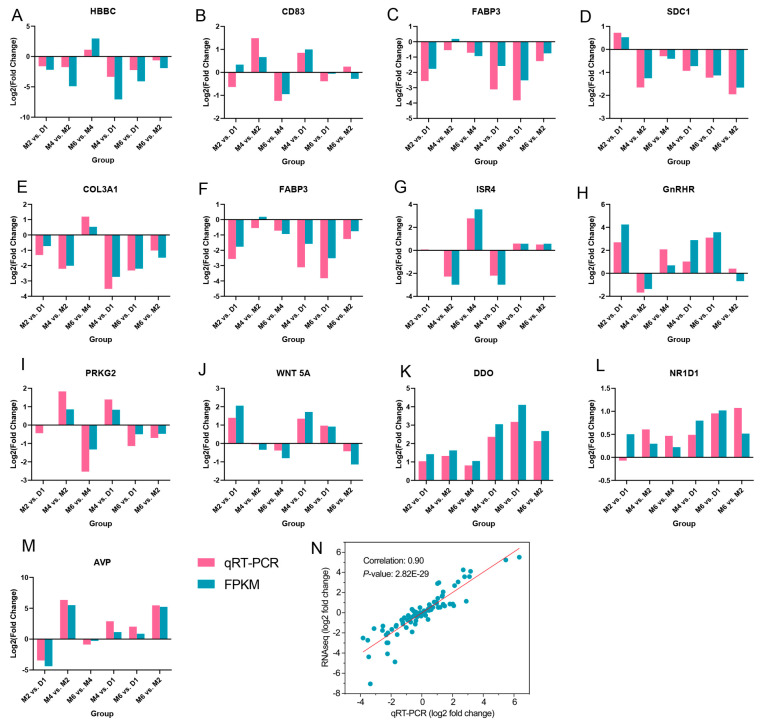
Quantitative validation of goat hypothalamus transcriptome data. (**A**–**M**): The *X*-axis represents different comparison groups, and the *Y*-axis represents log2(FoldChange) of qRT-PCR and RNA-seq. (**N**): The Correlation between qRT-PCR and RNA-seq (log2(FoldChange)).

To verify RNA-seq results, we have selected 13 DEGs for qRT-PCR validation. The log_2_(FoldChange) of RNA-Seq and qRT-PCR was consistent, and there was a strong correlation between the two sets of data (Figure 7), indicating that the transcriptome sequencing results were reliable.

## 3. Discussion

The hypothalamus plays a crucial role in regulating energy metabolism and reproductive function, significantly influencing sexual maturation and growth in mammals [25]. Precocious puberty leads to a more rapid generational turnover, reduces feeding costs, and shortens the reproductive cycle [26,27]. Studying the dynamic changes in hypothalamic transcriptome expression profiles during postnatal sexual development in goats is essential for understanding the molecular mechanisms of sexual maturation and reproductive physiology, and it provides valuable insights for goat breeding. Based on this, we analyzed the dynamic changes in serum hormone levels and hypothalamic transcriptome expression profiles across four postnatal developmental stages (D1, M2, M4, and M6) in female Jining grey goats.

The results indicated that the serum hormone levels in Jining grey goats exhibited distinct variations. Specifically, the levels of FSH, LH, and E2 were lowest during the D1 stage and significantly increased during the M2 stage. Previous studies have shown that lambs have the lowest serum levels of sex hormones, FSH, and LH immediately after birth [28]. Prior to puberty, as follicular development occurs, the levels of gonadal steroid hormones, luteinizing hormones, and follicle-stimulating hormones increase [29]. These findings align with our results. Furthermore, serum levels of LEP and IGF1 in goats during the M4 stage were significantly higher than in the D1 stage. This increase may be associated with the role of these hormones in sexual maturation and energy metabolism in animals [30].

Transcriptome analysis revealed that a total of 508 DEGs were identified at various stages of sexual development following birth in Jining grey goats. These DEGs can be distinctly categorized into four different expression patterns. We further compared the functional enrichment analysis results of DEGs across different comparison groups and expression patterns. Cluster 1 and the comparisons between M2 vs. D1, M4 vs. D1, and M6 vs. D1 exhibited significant enrichment in immune-related pathways, including the IL-17 signaling pathway, TGF-β signaling pathway, and antigen processing and presentation, among others. Following birth, goats acquire immunoglobulins through colostrum consumption, thus establishing their passive immunity [31]. As they mature, goats transition from passive to active immunity, a process that is implicated in sexual development postnatally [32,33]. Furthermore, the HPG axis and associated hormones play a role in regulating immune function [34,35]. In mammals, GnRH stimulates the expression of cytokines such as interleukin-2 (IL-2) and interferon gamma (IFN-γ), thereby promoting immune cell activation [36]. This study aligns with these findings, suggesting that postnatal sexual development and the immune system interact to regulate the growth and development of goats [37,38].

Additionally, circadian rhythms, neuroactive ligand receptor interaction pathways, and ECM receptor interaction pathways were significantly enriched. Research has demonstrated that these pathways are involved in regulating puberty in goats, sheep, pigs, and Brahman cattle [23,39,40]. NR1D1 has been identified as a key DEG within the circadian pathway and is implicated in regulating circadian balance and endocrine changes in goats and sheep [41,42,43]. NR1D1 can interact with the GnRH signaling pathway, contributing to the regulation of reproductive hormone synthesis and secretion [44], whereas the knockdown of NR1D1 reduces reproductive capability in mice [45]. In our study, NR1D1 expression in goat hypothalamic tissue increased progressively from D1 to M6, and showed a significant positive correlation with serum E2 levels. Additionally, genes like GnRH1 and the glucagon-like peptide 1 receptor (GLP1R) were significantly enriched in the neuroactive ligand–receptor interaction pathway. Research indicates that GnRH1 regulates sexual development by controlling the synthesis of pituitary LH and FSH [46,47,48], which in turn influences the release of gonadal steroid hormones. Furthermore, expression levels of GnRH1 in the hypothalamus significantly increased with the sexual maturity of goats [49]. This aligns with our findings, which revealed that GnRH1 expression rose considerably from D1 to M2 and was positively correlated with serum FSH and E2 levels. GLP1R, a G protein-coupled receptor present in various organs, including components of the gonadal axis [50], has also been studied for its role in reproduction. Research has shown that the deletion of GLP1R is associated with reproductive aging and fertility impairment in mice [51]. Moreover, GLP1 promotes GnRH secretion by binding to GLP1R in hypothalamic GnRH neurons in sheep [52]. GLP1R is also implicated in the regulation of food intake, insulin secretion, and energy homeostasis [53]. Changes in the body’s energy status feed back to the hypothalamus through signals from peripheral metabolic hormones such as leptin and insulin, thereby regulating growth and sexual development [54]. In this study, GLP1R levels in goats significantly increased from D1 to M4 and were positively correlated with serum IGF-1 levels, suggesting that GLP1R may play a crucial role in the sexual maturation of goats by promoting GnRH secretion or participating in energy metabolism. These findings indicate that differentially expressed genes across various developmental stages post-birth may significantly influence hormone secretion and energy metabolism during sexual development through these pathways.

Furthermore, the PPI analysis identified six key genes: BUB1, BUB1B, CENPE, BIRC5, KIF11, and DLGAP5. These genes are closely associated with cell proliferation and differentiation [55,56] and may influence the proliferation of goat hypothalamic cells by regulating the cell cycle pathway, thereby impacting the process of sexual maturity in goats.

This study provides important insights into the dynamic changes in hypothalamic tissue during the sexual maturation process in goats, but it still has certain limitations. First, a larger sample size and additional molecular biology experiments are needed to enhance the reliability and persuasiveness of the results. Then, since our analysis was conducted on Jining grey goats, it is necessary to verify the applicability of these findings to other goat breeds.

## 4. Materials and Methods

### 4.1. Sample Collection

A total of 20 female Jining grey goats were selected for this experiment from the Jining Grey Goat Breeding Farm (Jiaxiang County, Jining City, Shandong Province, China). The goats were divided into four age groups: 1-day-old group (neonatal, D1, n = 5), 2-month-old group (pre-puberty, M2, n = 5), 4-month-old group (sexual maturity, M4, n = 5), and 6-month-old group (breeding period, M6, n = 5). The selected goats were healthy, with no diseases. Within each group, their body condition was consistent, and none were in the estrus period. They were raised under identical conditions with ad libitum access to food and water. All goats were slaughtered on the same day. Following stunning via electric shock, the goats were promptly slaughtered. Serum and hypothalamic tissue samples were collected on the same day.

All hypothalamic samples were stored at −80 °C until further RNA extraction and analysis. Five milliliters of blood were collected from the jugular vein, left to stand for 1 h, and centrifuged at 3000× *g* for 10 min. The supernatant was placed in a centrifuge tube and stored at −80 °C for serum hormone index determination.

### 4.2. Serum Hormone Concentration Test

Serum concentrations of GnRH, FSH, LH, E2, P, LEP, and IGF-1 were measured using an enzyme-linked immunosorbent assay (ELISA). The ELISA kits used were goat-specific kits (MDBio, Qingdao, China).

### 4.3. RNA Extraction, Library Construction and Sequencing

Total RNA was extracted from 20 hypothalamic tissues using TRIzol reagent (Takara Bio, Dalian, China) according to the manufacturer’s protocol. The quality and concentration of RNA were assessed using Nanodrop 2000 (Thermo Scientific, Wilmington, DE, USA) and agarose gel electrophoresis. Subsequently, eligible RNA samples were selected for library construction. The removal of rRNA from the total RNA was accomplished using the Ribo-Zero™ kit (Illumina, San Diego, CA, USA). rRNA was fragmented into small fragments by adding a fragmentation buffer to the enriched RNA. The fragmented RNA was used as a template to synthesize the first cDNA by reverse transcription with 6 bp random hexamers. Buffer, dNTPs, DNA polymerase I, and RNase H were added to synthesize the second cDNA. The synthesized double-stranded cDNAs were subjected to PCR enrichment and finally purified by using AMPure XP microbeads (Beckman Coulter, Brea, CA, USA) to purify the PCR products to obtain a total of 20 libraries, which were subjected to paired-end 150 sequencing (PE150) using a NovaSeq 6000 sequencer (Illumina, San Diego, CA, USA).

### 4.4. Transcriptomic Sequencing Analysis

The quality evaluation of raw reads was performed using FastQC (v0.11.8). The filtering of reads containing adapters, poly-N sequences, or low quality was performed using Trimmomatic (v0.39), and the Q20, Q30, and GC contents of clean reads were calculated. Filtered high-quality clean reads were used for all subsequent analyses. A reference genome index was constructed using HISAT2 (v2.0.5), and clean reads were aligned to the goat reference genome (GCF_001704415.2_ARS1.2_genomic.fna.gz) using StringTie (v1.3.3b) to assemble the data.

### 4.5. Identification and Functional Analysis of DEGs

The calculation of mapped reads for each gene was conducted, and gene expression was quantified as the number of fragments per kilobase per million localized fragment transcripts (FPKM). Identifying differentially expressed genes among different comparison groups (M2 vs. D1, M4 vs. D1, M6 vs. D1, M4 vs. M2, M6 vs. M4, M6 vs. M2) was performed using the R software package DESeq2 (v1.20.0). The *p*-value was adjusted using the Benjamini and Hochberg method, and genes with a p.adj < 0.05 and|log2 Foldchange (FC)| > 1 were identified as DEGs. To gain deeper insights into the expression pattern of DEGs of six comparison groups, temporal clustering analysis was performed using the Mfuzz package [57]. The clusterProfiler software package (v3.10.1) was used for the GO enrichment analysis of DEGs with different expression modes [58], and KOBAS was used for the KEGG enrichment analysis of these DEGs [59]. *p* < 0.05 for GO terms and KEGG pathways were considered to be significantly enriched. Bubble and bar graphs were plotted using R software’s “ggplot2” package.

### 4.6. Protein–Protein Interaction Network Analysis

The PPI networks were obtained using the Search Tool for the Retrieval of Interacting Genes (STRING) database. DEGs are represented as nodes in the network, while the edges represent interactions between two DEGs. In this PPI network, interactions with a combined score exceeding 0.4 were considered reliable. A visual of the PPI networks involving DEGs was accomplished using Cytoscape. To visualize the PPI network, we employed the CytoHubba tool in Cytoscape and utilized various methods such as Degree, EPC, EcCentricity, MCC, and MNC to analyze key genes [60]. The MCODE plugin was used to analyze key modules within the PPI network, and the KEGG enrichment analysis of the genes in these modules was performed using KOBAS.

### 4.7. Validation of Differentially Expressed Genes

Fourteen DEGs were randomly selected for quantitative real-time PCR (qRT–PCR) validation. cDNA was synthesized from RNA extracted from the same batch as RNA-seq, employing a reverse transcription kit (Takara, Dalian, China). Subsequently, qRT-PCR was conducted on an LC96 instrument, utilizing primers designed using Primer Premier 5.0. The primers used are shown in Table S20. The reaction volume was 20 μL, comprising 2 μL of cDNA, 0.5 μL each of upstream and downstream primers (200 nM), 10 μL of SYBR (Takara, Dalian, China), and 7 μL of RNA-free water. The reaction was conducted employing a two-step method with the following conditions: 95 °C for 30 s, 95 °C for 10 s, 60 °C for 60 s, and 40 cycles. Glyceraldehyde-3-phosphate dehydrogenase (*GAPDH)* was employed as an internal reference gene to normalize the gene expression levels [61,62]. Relative expression levels were determined using the 2^−ΔΔCT^ method. There were 5 (n = 5) biological replicates.

### 4.8. Statistical Analysis

Data analysis was conducted using SPSS 22.0 (IBM Corp., Armonk, NY, USA). Intergroup variability for body weight, hypothalamic weight, and serum hormone levels was assessed using one-way ANOVA. Pearson correlations were employed to evaluate phenotypic data and the correlation between genes and phenotypic data. All experimental results are presented as mean ± standard error (mean ± SEM), with five biological replicates (n = 5). Statistical significance was set at *p* < 0.05. Graphs were plotted using GraphPad Prism 8.3 and R software (v4.2.1).

## 5. Conclusions

In this study, we explored the molecular regulatory mechanisms governing sexual maturation in goats through the integration of transcriptome data derived from the hypothalamus and serum hormone levels. The identified DEGs are predominantly associated with biological processes pertinent to reproduction, including circadian rhythm, the Wnt signaling pathway, and neuroactive ligand–receptor interactions. Furthermore, NR1D1, GnRH1, and GLP1R are likely to modulate sexual maturation via the regulation of hormonal secretion and energy metabolism in goats.

## Data Availability

The data presented in this study are openly available in NCBI at https://www.ncbi.nlm.nih.gov, accession number: GSE244004.

## Supplementary Materials

The following supporting information can be downloaded at https://www.mdpi.com/article/10.3390/ijms251810055/s1.

## References

[1] MacHugh D.E., Bradley D.G. (2001). Livestock genetic origins: Goats buck the trend. Proc. Natl. Acad. Sci. USA.

[2] Rumosa Gwaze F., Chimonyo M., Dzama K. (2009). Communal goat production in Southern Africa: A review. Trop. Anim. Health Prod..

[3] Miller B.A., Lu C.D. (2019). Current status of global dairy goat production: An overview. Asian-Australas. J. Anim. Sci..

[4] Parent A.S., Teilmann G., Juul A., Skakkebaek N.E., Toppari J., Bourguignon J.P. (2003). The timing of normal puberty and the age limits of sexual precocity: Variations around the world, secular trends, and changes after migration. Endocr. Rev..

[5] Ojeda S.R., Lomniczi A., Mastronardi C., Heger S., Roth C., Parent A.S., Matagne V., Mungenast A.E. (2006). Minireview: The neuroendocrine regulation of puberty: Is the time ripe for a systems biology approach?. Endocrinology.

[6] Scott C.J., Rose J.L., Gunn A.J., McGrath B.M. (2018). Kisspeptin and the regulation of the reproductive axis in domestic animals. J. Endocrinol..

[7] Flament-Durand J. (1980). The hypothalamus: Anatomy and functions. Acta Psychiatr. Belg..

[8] Swanson L.W. (2000). Cerebral hemisphere regulation of motivated behavior. Brain Res..

[9] Maeda K., Ohkura S., Uenoyama Y., Wakabayashi Y., Oka Y., Tsukamura H., Okamura H. (2010). Neurobiological mechanisms underlying GnRH pulse generation by the hypothalamus. Brain Res..

[10] Herbison A.E. (2016). Control of puberty onset and fertility by gonadotropin-releasing hormone neurons. Nat. Rev. Endocrinol..

[11] Liu Z., Fu S., He X., Dai L., Liu X., Narisu, Shi C., Gu M., Wang Y., Manda (2023). Integrated Multi-Tissue Transcriptome Profiling Characterizes the Genetic Basis and Biomarkers Affecting Reproduction in Sheep (*Ovis aries*). Genes.

[12] Zhu J., Chen F., Luo L., Wu W., Dai J., Zhong J., Lin X., Chai C., Ding P., Liang L. (2021). Single-cell atlas of domestic pig cerebral cortex and hypothalamus. Sci. Bull..

[13] Liu M., Zhang C., Chen J., Xu Q., Liu S., Chao X., Yang H., Wang T., Muhammad A., Schinckel A.P. (2024). Characterization and analysis of transcriptomes of multiple tissues from estrus and diestrus in pigs. Int. J. Biol. Macromol..

[14] Hou B., Mao M., Dong S., Deng M., Sun B., Guo Y., Li Y., Liu D., Liu G. (2023). Transcriptome analysis reveals mRNAs and long non-coding RNAs associated with fecundity in the hypothalamus of high-and low-fecundity goat. Front. Vet. Sci..

[15] Zhang Z., Sui Z., Zhang J., Li Q., Zhang Y., Xing F. (2022). Transcriptome Sequencing-Based Mining of Genes Associated with Pubertal Initiation in Dolang Sheep. Front. Genet..

[16] Fortes M.R., Nguyen L.T., Weller M.M., Cánovas A., Islas-Trejo A., Porto-Neto L.R., Reverter A., Lehnert S.A., Boe-Hansen G.B., Thomas M.G. (2016). Transcriptome analyses identify five transcription factors differentially expressed in the hypothalamus of post-versus prepubertal Brahman heifers. J. Anim. Sci..

[17] Li Q., Pan X., Li N., Gong W., Chen Y., Yuan X. (2021). Identification of Circular RNAs in Hypothalamus of Gilts during the Onset of Puberty. Genes.

[18] Chu M.X., Jiao C.L., He Y.Q., Wang J.Y., Liu Z.H., Chen G.H. (2007). Association between PCR-SSCP of bone morphogenetic protein 15 gene and prolificacy in Jining Grey goats. Anim. Biotechnol..

[19] Miao X., Luo Q., Qin X. (2016). Genome-wide transcriptome analysis in the ovaries of two goats identifies differentially expressed genes related to fecundity. Gene.

[20] National Committee for Livestock and Poultry Genetic Resources (2011). Animal Genetic Resources in China: Sheep and Goats.

[21] Shi Y., Wang S., Bai S., Huang L., Hou Y. (2015). Postnatal ovarian development and its relationship with steroid hormone receptors in JiNing Grey goats. Anim. Reprod. Sci..

[22] Gao X., Ren C., Zhang W., Fang F. (2023). An integrated analysis of mRNAs and lncRNAs in goat’s hypothalamus to explore the onset of puberty. Reprod. Domest. Anim..

[23] Su F., Guo X., Wang Y., Wang Y., Cao G., Jiang Y. (2018). Genome-Wide Analysis on the Landscape of Transcriptomes and Their Relationship with DNA Methylomes in the Hypothalamus Reveals Genes Related to Sexual Precocity in Jining Gray Goats. Front. Endocrinol..

[24] Cao G.L., Feng T., Chu M.X., Di R., Zhang Y.L., Huang D.W., Liu Q.Y., Hu W.P., Wang X.Y. (2015). Subtraction suppressive hybridisation analysis of differentially expressed genes associated with puberty in the goat hypothalamus. Reprod. Fertil. Dev..

[25] Yang F., Zhao S., Wang P., Xiang W. (2023). Hypothalamic neuroendocrine integration of reproduction and metabolism in mammals. J. Endocrinol..

[26] Wang W., Wang Y., Liu Y., Cao G., Di R., Wang J., Chu M. (2023). Polymorphism and expression of GLUD1 in relation to reproductive performance in Jining Grey goats. Arch. Anim. Breed..

[27] Valasi I., Chadio S., Fthenakis G.C., Amiridis G.S. (2012). Management of pre-pubertal small ruminants: Physiological basis and clinical approach. Anim. Reprod. Sci..

[28] Georgieva R.I., Bulahbel S., Georgiev H.G. (1994). Patterns of variations in FSH, LH and 17beta-estradiol during the postnatal development of sheep. Theriogenology.

[29] Rawlings N.C., Evans A.C., Honaramooz A., Bartlewski P.M. (2003). Antral follicle growth and endocrine changes in prepubertal cattle, sheep and goats. Anim. Reprod. Sci..

[30] Ferraz M.V.C., Santos M.H., Oliveira G.B., Polizel D.M., Barroso J.P.R., Nogueira G.P., Gouvea V.N., Carvalho P.H.V., Biava J.S., Ferreira E.M. (2023). Effect of growth rates on hormonal and pubertal status in Nellore heifers early weaned. Trop. Anim. Health Prod..

[31] Zhou A., Liu G., Jiang X. (2023). Characteristic of the components and the metabolism mechanism of goat colostrum: A review. Anim. Biotechnol..

[32] Struff W.G., Sprotte G. (2007). Bovine colostrum as a biologic in clinical medicine: A review. Part I: Biotechnological standards, phar-macodynamic and pharmacokinetic characteristics and principles of treatment. Int. J. Clin. Pharmacol. Ther..

[33] Guedes M.T., Zacharias F., Couto R.D., Portela R.W., Santos L.C., Santos S.C., Pedroza K.C., Peixoto A.P., López J.A., Mendonça-Lima F.W. (2010). Maternal transference of passive humoral immunity to *Haemonchus contortus* in goats. Vet. Immunol. Immunopathol..

[34] Segner H., Verburg-van Kemenade B.M.L., Chadzinska M. (2017). The immunomodulatory role of the hypothalamus-pituitary-gonad axis: Proximate mechanism for reproduction-immune trade offs?. Dev. Comp. Immunol..

[35] Grossman C.J. (1985). Interactions between the gonadal steroids and the immune system. Science.

[36] Weigent D.A., Blalock J.E. (1995). Associations between the neuroendocrine and immune systems. J. Leukoc. Biol..

[37] Vitzthum V.J. (2009). The ecology and evolutionary endocrinology of reproduction in the human female. Am. J. Phys. Anthropol..

[38] Angioni S., Petraglia F., Genezzani A.R. (1991). Immune-neuroendocrine correlations: A new aspect in human physiology. Acta Eur. Fertil..

[39] Chu Q., Zhou B., Xu F., Chen R., Shen C., Liang T., Li Y., Schinckel A.P. (2017). Genome-wide differential mRNA expression profiles in follicles of two breeds and at two stages of estrus cycle of gilts. Sci. Rep..

[40] Nguyen L.T., Lau L.Y., Fortes M.R.S. (2022). Proteomic Analysis of Hypothalamus and Pituitary Gland in Pre and Postpubertal Brahman Heifers. Front. Genet..

[41] Lincoln G.A., Johnston J.D., Andersson H., Wagner G., Hazlerigg D.G. (2005). Photorefractoriness in mammals: Dissociating a seasonal timer from the circadian-based photoperiod response. Endocrinology.

[42] Xiao Y., Zhao L., Li W., Wang X., Ma T., Yang L., Gao L., Li C., Zhang M., Yang D. (2021). Circadian clock gene BMAL1 controls testosterone production by regulating steroidogenesis-related gene transcription in goat Leydig cells. J. Cell Physiol..

[43] Dardente H., Fustin J.M., Hazlerigg D.G. (2009). Transcriptional feedback loops in the ovine circadian clock. Comp. Biochem. Physiol. Mol. Integr. Physiol..

[44] Cho H., Zhao X., Hatori M., Yu R.T., Barish G.D., Lam M.T., Chong L.W., DiTacchio L., Atkins A.R., Glass C.K. (2012). Regulation of cir-cadian behaviour and metabolism by REV-ERB-α and REV-ERB-β. Nature.

[45] Chomez P., Neveu I., Mansén A., Kiesler E., Larsson L., Vennström B., Arenas E. (2000). Increased cell death and delayed development in the cerebellum of mice lacking the rev-erbA(alpha) orphan receptor. Development.

[46] Ma Y., Juntti S.A., Hu C.K., Huguenard J.R., Fernald R.D. (2015). Electrical synapses connect a network of gonadotropin releasing hormone neurons in a cichlid fish. Proc. Natl. Acad. Sci. USA.

[47] Schally A.V., Arimura A., Kastin A.J., Matsuo H., Baba Y., Redding T.W., Nair R.M., Debeljuk L., White W.F. (1971). Gonadotropin-releasing hormone: One polypeptide regulates secretion of luteinizing and follicle-stimulating hormones. Science.

[48] Avet C., Denoyelle C., L‘Hôte D., Petit F., Guigon C.J., Cohen-Tannoudji J., Simon V. (2018). GnRH regulates the expression of its receptor accessory protein SET in pituitary gonadotropes. PLoS ONE.

[49] Yamamoto T., Nakahata Y., Soma H., Akashi M., Mamine T., Takumi T. (2004). Transcriptional oscillation of canonical clock genes in mouse peripheral tissues. BMC Mol. Biol..

[50] Jensterle M., Janez A., Fliers E., DeVries J.H., Vrtacnik-Bokal E., Siegelaar S.E. (2019). The role of glucagon-like peptide-1 in reproduction: From physiology to therapeutic perspective. Hum. Reprod. Update.

[51] Nedyalkova M., Robeva R., Romanova J., Yovcheva K., Lattuada M., Simeonov V. (2024). In silico screening of potential agonists of a glucagon-like peptide-1 receptor among female sex hormone derivatives. J. Biomol. Struct. Dyn..

[52] Arbabi L., Li Q., Henry B.A., Clarke I.J. (2021). Glucagon-like peptide-1 control of GnRH secretion in female sheep. J. Endocrinol..

[53] Holst J.J. (2007). The physiology of glucagon-like peptide 1. Physiol. Rev..

[54] Garza V., West S.M., Cardoso R.C. (2023). Review: Gestational and postnatal nutritional effects on the neuroendocrine control of puberty and subsequent reproductive performance in heifers. Animal.

[55] Toledo C.M., Herman J.A., Olsen J.B., Ding Y., Corrin P., Girard E.J., Olson J.M., Emili A., DeLuca J.G., Paddison P.J. (2014). BuGZ is required for Bub3 stability, Bub1 kinetochore function, and chromosome alignment. Dev. Cell.

[56] Yao X., Abrieu A., Zheng Y., Sullivan K.F., Cleveland D.W. (2000). CENP-E forms a link between attachment of spindle microtubules to kinetochores and the mitotic checkpoint. Nat. Cell Biol..

[57] Kumar L., Futschik M.E. (2007). Mfuzz: A software package for soft clustering of microarray data. Bioinformation.

[58] Yu G., Wang L.G., Han Y., He Q.Y. (2012). clusterProfiler: An R package for comparing biological themes among gene clusters. Omics.

[59] Bu D., Luo H., Huo P., Wang Z., Zhang S., He Z., Wu Y., Zhao L., Liu J., Guo J. (2021). KOBAS-i: Intelligent prioritization and exploratory visualization of biological functions for gene enrichment analysis. Nucleic Acids Res..

[60] Chin C.H., Chen S.H., Wu H.H., Ho C.W., Ko M.T., Lin C.Y. (2014). cytoHubba: Identifying hub objects and sub-networks from complex interactome. BMC Syst. Biol..

[61] Cleal J.K., Shepherd J.N., Shearer J.L., Bruce K.D., Cagampang F.R. (2014). Sensitivity of housekeeping genes in the suprachiasmatic nucleus of the mouse brain to diet and the daily light-dark cycle. Brain Res..

[62] Otto E., Köhli P., Appelt J., Menzel S., Fuchs M., Bahn A., Graef F., Duda G.N., Tsitsilonis S., Keller J. (2020). Validation of reference genes for expression analysis in a murine trauma model combining traumatic brain injury and femoral fracture. Sci. Rep..

