# Supplementary material for: The Transcriptome Characterization of the Hypothalamus and the Identification of Key Genes during Sexual Maturation in Goats
Source: Int J Mol Sci. 2024 Sep 19;25(18):10055. doi: 10.3390/ijms251810055 (PMC11432450; doi:10.3390/ijms251810055)
Supplement: Supplementary file 1 [file ijms-25-10055-s001.zip › Supplementary Figure.pdf]

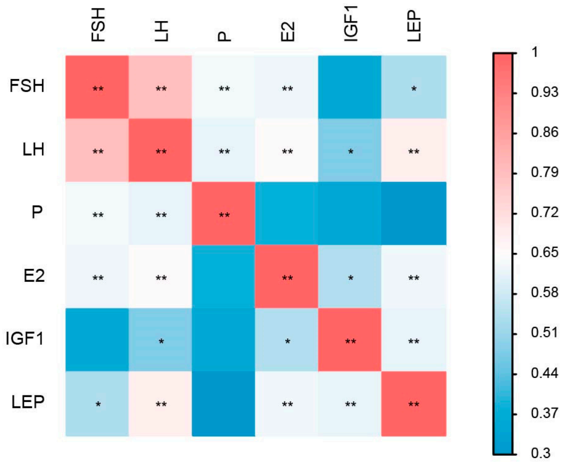

Figure S1. Correlation analysis of serum hormone levels of Jining grey goats. * indicates that the correlation between the two traits has reached a significant level ($P < 0.05$), ** indicates that the correlation between the two traits has reached an extremely significant level ($P < 0.01$). follicle-stimulating hormone, FSH; luteinizing hormones, LH, progesterone, P; estradiol, E2; Insulin-like growth factor 1, IGF-1; Leptin, LEP.

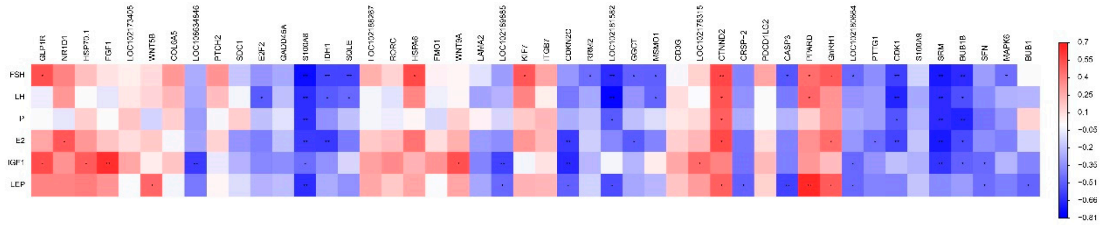

Figure S2. Correlation analysis of differentially expressed genes (DEGs) involved in reproductive pathways at different developmental stages of Jining grey goats and serum hormone levels. * indicates that the correlation between the two traits has reached a significant level (*P* < 0.05), ** indicates that the correlation between the two traits has reached an extremely significant level (*P* < 0.01).

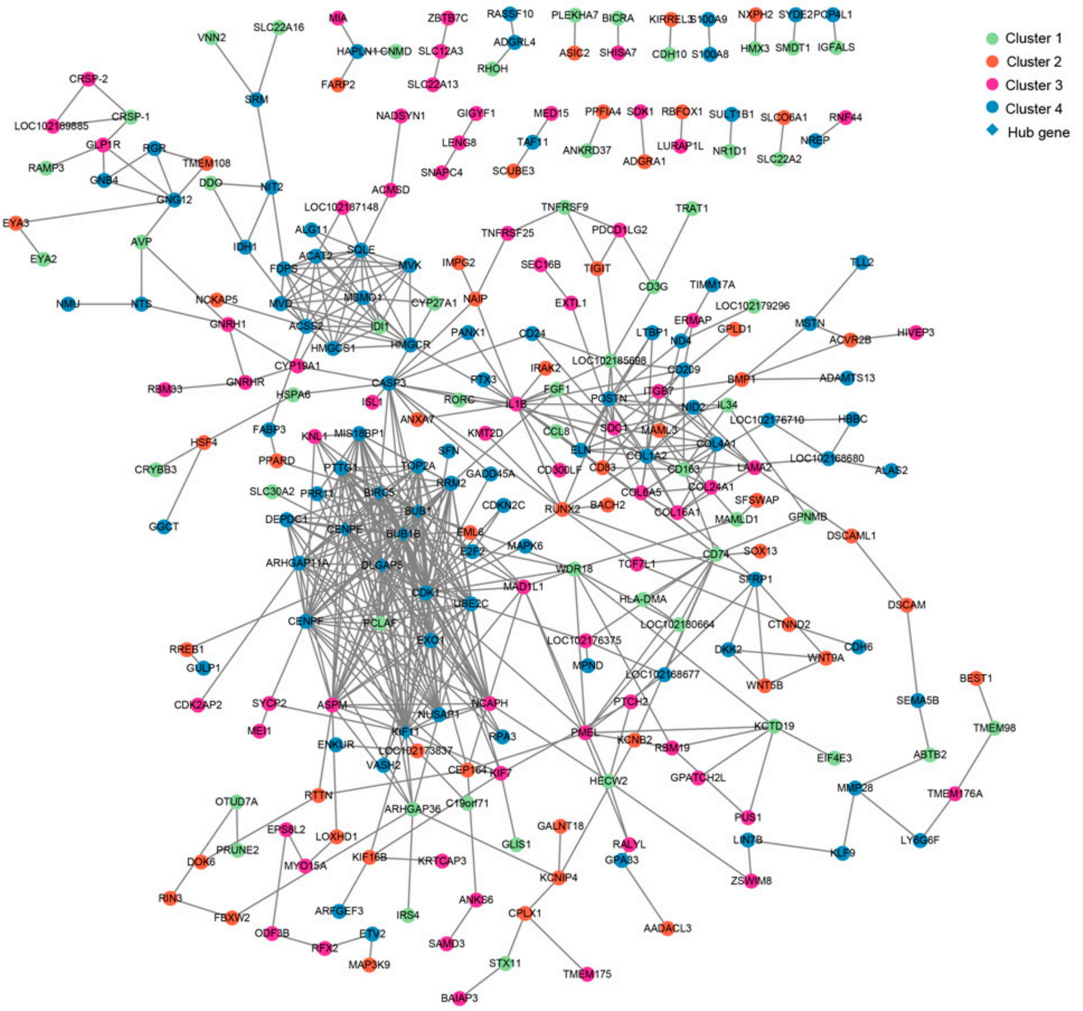

Figure S3. Protein-protein interaction (PPI) networks of DEGs in different developmental hypothalamic transcriptomes. The network consists of 267 nodes and 620 edges. The red nodes represent DEGs that were predicted by all five analysis methods (edge percolated component (EPC); maximum neighborhood component (MNC); maximal clique centrality (MCC); degree; eccentricity). Different colors indicate DEGs with different expression patterns.
